# Allergic diseases in the elderly: biological characteristics and main immunological and non-immunological mechanisms

**DOI:** 10.1186/s12948-017-0059-2

**Published:** 2017-02-03

**Authors:** Maria Teresa Ventura, Nicola Scichilone, Roberto Paganelli, Paola Lucia Minciullo, Vincenzo Patella, Matteo Bonini, Giovanni Passalacqua, Carlo Lombardi, Livio Simioni, Erminia Ridolo, Stefano R. Del Giacco, Sebastiano Gangemi, Giorgio Walter Canonica

**Affiliations:** 1 Interdisciplinary Department of Medicine, Unit of Geriatric Immunoallergology, University of Bari Medical School, Bari, Italy; 2 DIBIMIS, University of Palermo, Palermo, Italy; 3 Laboratory of Immunology and Allergy, Department of Medicine and Sciences of Aging, University of G. d’Annunzio, Chieti, Italy; 4 Division and School of Allergy and Clinical Immunology, Department of Clinical and Experimental Medicine, University Hospital of Messina, Messina, Italy; 5 Division of Allergy and Clinical Immunology, Department of Medicine, Battipaglia Hospital, Battipaglia, Salerno, Italy; 6 School of Allergy and Clinical Immunology, University of Naples Federico II, Naples, Italy; 7 National Heart and Lung Institute (NHLI), Imperial College London & Royal Brompton Hospital, London, UK; 8 Allergy and Respiratory Diseases, IRCCS San Martino-IST-University of Genoa, Genoa, Italy; 9 Departmental Unit of AllergologyClinical Immunology & Pneumology, Fondazione Poliambulanza Hospital, Brescia, Italy; 10 Department of Medicine, Allergy Service, ULSS 2 Feltre, Belluno, Italy; 11 Experimental and Clinical Medicine, University of Parma, Parma, Italy; 12 Department of Medical Sciences “M. Aresu”, University of Cagliari, Cagliari, Italy

**Keywords:** Aging, Elderly, Allergy, Conjunctivitis, Asthma, Immunotherapy, Urticaria, Drug reaction, Anaphylaxis, Food allergy

## Abstract

Life expectancy and the number of elderly people are progressively increasing around the world. Together with other pathologies, allergic diseases also show an increasing incidence in geriatric age. This is partly due to the growing emphasis on a more accurate and careful diagnosis of the molecular mechanisms that do not allow to ignore the real pathogenesis of many symptoms until now unknown, and partly to the fact that the allergic people from 20 years ago represent the elderly population now. Moreover, environmental pollution predisposes to the onset of allergic asthma and dermatitis which are the result of internal pathologies more than the expression of allergic manifestations. At the same time the food contamination permits the onset of allergic diseases related to food allergy. In this review we provide the state of the art on the physiological changes in the elderly responsible for allergic diseases, their biological characteristics and the major immunological and extra immunological mechanisms. Much emphasis is given to the management of several diseases in the elderly, including anaphylactic reactions. Moreover, some new features are discussed, such as management of asthma with the support of physical activity and the use of the AIT as prevention of respiratory diseases and for the purpose of a real and long lasting benefit. The mechanisms of adverse reactions to drugs are also discussed, due to their frequency in this age, especially in polytherapy regimens. Study of the modifications of the immune system is also of great importance, as regards to the distribution of the lymphocytes and also the presence of a chronic inflammatory disease related to the production of cytokines, especially in prevision of all the possible therapies to be adopted to allow an active and healthy aging.

## Background

The aging population in Europe and all over the world implicates increasing social problems and a specific scientific support is by now as required in order to ensure what is called a “successful aging”. In fact, epidemiological data attest always more the need for a healthy aging to contain the increased economic burden for assistance of a growing number of people over 65 in condition of “fragility”. Aging is an unavoidable, universal, complex process characterized by progressive loss of functional reserves and reduction of the capability to adapt to the environment. This biological phenomenon derives from an interaction between genetic and environmental factors. Healthy lifestyle and favorable environment allow genetically predisposed subjects to reach extreme longevity and maintain acceptable health status and auto-sufficiency [1].

Disfunctions of the immune system are the basis of many disabling diseases that occur during aging, including neurodegenerative and cardiovascular diseases, whose appearance is due to the state of proinflammatory, also called “inflamm-aging”, that is typical of the elderly [2]. The mechanisms that enable a “successful” aging are related to certain physical and immunologic conditions. This is due to multiple factors, including the increased pollution; moreover the allergic pediatric population that was born 50–60 years ago has reached the age of senescence. Therefore, diseases such as food allergy, urticaria, allergic reactions to drugs, and anaphylaxis are becoming more frequent in the elderly population [3].

The purpose of this work is to provide the state of the art on the physiological changes in the elderly responsible for allergic diseases, their biological characteristics and the major immunological and extra immunological mechanisms.

### Lymphocyte subpopulations in the elderly

Most information on lymphocyte subpopulations and their changes occurring with age derive from the cytofluorimetric analysis using monoclonal antibodies directed to surface antigens of gated lymphoid cells collected from peripheral venous blood. It is well known that the total number of lymphocytes—the main circulating subset of leukocytes in the first years of life declines since young adulthood and then reaches very low numbers from age 60 onwards. And it is also known that usually about 2% of all lymphocytes appear to recirculate in the blood, while the remaining are resident in gut and spleen or circulate through all the lymphoid system (lymph nodes, mucosal aggregates, thoracic duct, tonsils etc.). Therefore our assumption is that the blood circulating 2% should reflect the proportion of lymphocyte subpopulations really present in the entire body. This can be true for some (e.g. γ/δ T cells, NK T cells, CD8 αα, and many others), but not all subsets, because some are strictly compartmentalized mainly to the mucosa. The main lymphocyte subpopulations discussed briefly here will be T subsets, B cells and innate immunity.

At birth high numbers of B cells are present, and low NK cells, and they will change in opposite directions during the individual’s lifetime. T cells are generated through thymic selection and the entire repertoire is established by the 10th year of life, while the thymus will undergo progressive involution from the age of 30. T cells represent the major subset (55–70% of all lymphocytes) in young people and so remain until late age. However the two main T cell subsets, identified by the coreceptor molecules CD4 and CD8, show different changes with ageing, with a progressive increase of CD8+ cells with advancing age.

CD4+ and CD8+ peripheral blood T lymphocytes show mutually exclusive expression of CD45RA or CD45R0, two isoforms of the common leukocyte antigen that help recognize respectively virgin/unprimed and memory/activated T cells [4]. Changes in the subsets have been observed with age: at birth 95–99% of the CD3+ T lymphocytes expressed the CD45RA isoform, while an increase of CD45R0+ cells was observed until 30 years. Nonetheless, in very old age a proportion of CD45RA+ among CD4+ cells was still present (about 20%) and even more in CD8+ lymphocytes (up to 40–50%). These data originate from very selected subjects without chronic common pathologies or taking drugs able to affect the blood subpopulations. The reverse “frail” or disabled condition can lead to different observations, with many older subjects showing many more CD8+ and “memory” phenotypes. These considerations lead to analyze the presence and role of thyme in older age. The generation of new T cells is indeed crucial to maintain a functional immune system, and lymphocytes defined “recent thymic emigrants”, as well as the detection of the so called “T cell receptor rearrangement excision circles (TREC)”, have been studied [5], leading to the conclusion that new T cells are produced even in old age, although in very low numbers. Molecules able to stimulate thymic production include cytokines like interleukin (IL)-7, IL-15 and others. The level of expression of the interleukin 7 receptor (IL-7R) gene in blood has recently been found to be associated with familial longevity and healthy ageing [6]. IL-7R is crucial for T cell development and important for immune competence.

The increased CD4:CD8 ratios in the elderly were significantly lower in Cytomegalovirus (CMV)-seropositive individuals, that in our experience represent >98% of aged subjects. This study also showed a lower naïve and a larger late-differentiated compartment of CD8+ αβ T-cells. Longitudinal studies suggest that accumulation of these cells is a dynamic process as an age-associated change [7].

A coincevable hypothesis is that these events are at least partly due to the effects of the maintenance of essential immune surveillance against persistent viral infections, notably CMV, which may exhaust the immune system over time, as shown in the NONA immune longitudinal study [4]. From this and other analysis an immune risk profile (IRP) was identified, defined by an inverted CD4/CD8 ratio and associated with persistent cytomegalovirus infection and increased numbers of CD8+ CD28− cells, which led to a relative 6-year survival in NONA individuals.

As regards the functional characteristics of these T cell compartments in the elderly, exhausted or non-functional CD8+ cells have been described in several studies. T cell clones (TCC) of two healthy centenarians were studied and compared them with TCC obtained by three young normal subjects: totally 180TCC were analyzed. In young donors, 35TCC were CD4+, 56 CD8+ and two were alpha beta +CD4−CD8− (double negative). In centenarians, 46CD4+TCC, 38CD8+, 2CD4+CD8+ (double positive) and 1 gamma delta+ double negative were obtained [8]. The cytokine analysis showed that the Th profiles of CD8+TCC are nearly overlapping in two groups, whereas a major shift from a Th1 to a Th0 pattern is shown by CD4+TCC.

It is well known that T cells from aged subjects have a reduced ability to produce IL-2, but other functions seem to be upregulated, since IL-1, IL-4, IL-6 and TNFα production are increased. These cytokines are known to control B cell differentiation, through isotype switch and Ig production. A significant age-related increase of the serum level of immunoglobulin classes (IgG and IgA but not IgM) and IgG subclasses (IgG1, 2 and 3, but not IgG4) is detected [9]. An age-related decrease of circulating B cells occurs in parallel. The impairment of primary responses to immunization, and other aspects of humoral immunity, including mucosal responses, autoantibody production led to the hypothesis of a complex derangement of B cell function and/or compartmentalization with age. Unusual IgD(−)CD27(−) double-negative B cell population was described in the elderly [10]. Most of these were less functional memory B cells, so it is speculated these were also late memory or exhausted cells that have down-modulated the expression of CD27 and filled the immunologic space in the elderly.

Finally, also adaptive immunity undergoes severe deterioration with age and represents the main problem in the elderly. Evidence accumulated over the last decade supports the hypothesis that aging also has a profound impact on innate immunity, including reduced or dysfunctional NK lymphocytes, which in turn markedly affects the health and longevity of older people [11].

### The role of cytokines in the elderly

Aging is a dynamic process characterized by a continuous remodeling sustained by DNA repair, apoptosis, immune response, oxidative stress and inflammation. To reach extreme longevity, it is necessary for all these mechanisms to interact in an efficient way. The theory of inflammaging focuses on the activation of a subclinical, chronic low-grade inflammation that occurs with aging. It is a manifestation of immunosenescence, which is the sum of changes affecting the functionality of the immune system in elderly.

A major contributor of inflammaging is the antigenic load of persistent infection which starts from intrauterine life and continues for an entire lifespan. The pro-inflammatory phenotype of senescent cells protects against infectious diseases but, at the same time, contributes to inflammaging, as large quantities of pro-inflammatory cytokines are produced/released in order to repair tissue damage [12]. Many age-related diseases share an inflammatory pathogenesis; moreover, the activation of inflammatory pathways appears to be involved in the pathophysiology of sarcopenia and frailty. Long-lived people, especially centenarians, seem to cope with chronic subclinical inflammation through an anti-inflammatory response, called therefore “anti-inflammaging” [13]. We believe that if inflammaging is a key to understand aging, anti-inflammaging may be one of the secrets of longevity.

Circulating cytokines are involved both in inflammation and anti-inflammation, and are the expression of a system that involves genes and environment.

#### Proinflammatory cytokines in aging

The most relevant proinflammatory cytokines involved in aging processes are interleukin (IL)-1, IL-6, tumor necrosis factor (TNF) and interferon (IFN)-γ. High levels of these cytokines are associated with increased risk of morbidity and mortality in the older subject. In particular, IL-1 is involved in the alteration of nutritional status, in cognitive decline, and development of Alzheimer’s disease [14] and as a risk factor for depressive symptoms in aged people [15]. IL-6 and TNF serum levels influence the onset of frailty, poor physical performance, loss of muscle strength, cognitive decline, cerebrovascular and cardiovascular events [14, 16]. These cytokines are also closely linked to the genesis of cancers (IL-6), to cardiac remodeling in heart failure [14] and to the risk of community-acquired pneumonia requiring hospitalization (both IL-6 and TNF) [17].

An increase of intracellular IFN-γ, together with other type 1 cytokines, within three CD8+ T subsets (naïve, effector/cytotoxic and memory) was observed in aged subjects [18]. Conversely, the % age of IFN-γ positive cells significantly decreased in a virgin CD4+ subset as well as in activated/memory T cells from old subjects [19]. However, in a study on nonagenarians, IFN-γ plasma levels are unmodified compared to young controls, suggesting a substantial maintenance of relevant T cell functions [20].

Another potent proinflammatory cytokine that plays a relevant role in aging is IL-18. A recent study showed that IL-18 is a predictor of mortality for heart failure in a group of octogenarians. A study of follow-up (of mean 2.4 years) underlighted that subjects who died for heart failure had higher serum values of IL-18 compared with those still alive [21]. IL-18 serum levels increase with ageing [22], but at the same time longevity is characterized by a reduced incidence of ischemic events.

Our group evaluated serum levels of IL-18 and IL-18 binding protein (IL-18BP) in healthy centenarians, younger healthy controls and patients with chronic ischemic cardiac disease. Centenarians showed significantly higher levels of IL-18 and IL-18BP compared to each control group. Elevated IL-18 levels were also present in patients with chronic ischemic syndrome; centenarians exhibited a lower level of free IL-18 than chronic ischemic patients. These results indicate that inactivation of IL-18 by IL-18BP may explain the apparent paradox of high serum levels of IL-18 in centenarians, without signs of vascular diseases [23].

Significant increased IL-12 serum levels were found in nonagenarians compared to young controls; this might be related to the increase of NK cell functions characterizing aging processes [20]. Different data were shown by other authors [24] who found that age was not correlated with Toll-like receptor (TLR)-mediated IL-12 and IL-23 production after stimulation of whole blood cells. In contrast, poor nutritional status and frailty and not age by itself were associated with decreased IL-12 and IL-23 production. These results in frail, elderly patients could account for their susceptibility to many pathogens [24].

In a study of our group centenarians subjects showed significantly higher IL-15 levels compared to both young and old controls [25]. These findings may explain, at least in part, the characteristic increase of memory cells in elderly subjects and may lead to a better understanding of the capacity of adaptation to the environment of centenarians, defending themselves from infections through immune-inflammatory responses.

We also found increased IL-22 serum levels in older patients with chronic heart failure [26] and in healthy centenarians [27]. In particular, only the II and III NYHA class had IL-22 values significantly higher than the controls, whereas there was no difference between the IL-22 levels of NYHA class IV and the controls [26]. The reason may be the decline of immune function in patients with chronic heart failure. In fact we can hypothesize that the reduction of IL-22 levels with the progression of NYHA class can be responsible of the impaired ability of these patients to respond to infections, as IL-22 has anti-microbial properties [26]. Therefore, it is likely that the pro-inflammatory condition sustained by IL-22 is protective against infection, promoting the longevity of these subjects [27]. It might be speculated that under certain circumstances the activity of this cytokine against infection may be more relevant to survival than its potential negative impact on inflammation.

The data on IL-2 in long-lived people are controversial. An age-related decrease in IL-2 production by stimulated PBMC or lymphocytes and IL-2 serum levels has been reported [28–33]. Other authors found that intracellular production of IL-2 showed a progressive age-dependent increase in CD8+ T cells [18]. However, in other studies, serum IL-2 levels were unmodified in healthy aged people [34] and centenarians [20].

#### Anti-inflammatory cytokines in aging

Among anti-inflammatory cytokines IL-10 plays a central role. High levels of this cytokine in whole blood samples were found to be associated with successful aging and, particularly, with a markedly reduced risk of death from a cardiovascular event in aged people [16], but diminished resistance to infectious diseases [35]. High levels of IL-10 have been found in centenarians; this condition may result in protection from cancer [36].

IL-10 production is tightly regulated and several single nucleotide polymorphisms controlling production have been described. IL-10 low-producer genotypes seem to play a particular role in susceptibility to inflammatory diseases, together with age-related ones, whereas IL-10 high producer genotypes are involved in longevity [37].

Studies on IL-10 polymorphisms showed different results between males and females [reviewed in 38]. For this reason it seems that gender is a major variable in the genetics of longevity and that men and women probably follow different strategies to reach it. Discordant results on the association between IL-10 polymorphisms and longevity have been obtained in other populations. Therefore, cytokine/longevity associations have a population-specific component, being affected by the population-specific gene pool as well as by gene-environment interactions, behaving as survival rather than longevity genes [39].

IL1-Ra can be considered a significant predictor of mortality in elderly. A prospective population-based study, including 285 nonagenarians, demonstrated that plasma levels of IL-1Ra were higher in subjects who died during a 4-year follow-up than in those who survived [40]. However, another study on 1131 elderly Italians showed a significant age-related increase of IL-1Ra plasma levels. In long-lived individuals, this seems to be a safeguard mechanism to cope with the age-associated increased inflammatory state [41].

Contrasting data emerge from studies about the role of IL-4 on aged and long-lived people. Some authors [20] found a reduction of IL-4 serum levels and an increase of IL-13 in a group of nonagenarians, mirroring the maintenance of some effector mechanisms of the immune response in advanced ages. On the contrary, other authors [18], found that type 2 intracellular cytokines, including IL-4, increased within virgin, memory and effector/cytotoxic CD8+ T cells in aged people.

Another study determined IL-4 production in three CD4+ T-subsets—virgin, activated/memory, and effector/memory—at different ages. IL-4 positive cells appeared to increase, with age. In particular, IL-4 positive cells significantly increased in activated/memory T cells from nonagenarian subjects. The authors also found a statistically significant decreased ratio of IFN-γ/IL-4 within activated/memory T cells in old people in comparison with young people. These data suggest a dynamic shift towards an increased role of type 2 cytokines and a diminished role of type 1 cytokines in human ageing. However, it is important to remember that this phenomenon, which could be interpreted as a compensatory mechanism to the increased pro-inflammatory status, occurs within a background where type 1 cytokines are quantitatively dominant [19].

Also data reported on correlation between TGF-β levels and age are contrasting. A study reported serum TGF-β1 levels inversely associated with age [42]. However, high levels of TGF-β have been found in a group of octogenarians and nonagenarians [43] and in two different groups of centenarians [36, 44]. This condition suggests that TGF-β could both counteract and counterbalance the harmful effects of inflammaging and may result in protection from cancer. A recent study did not detect any differences in serum levels of TGF-β and TGF-β mRNA levels from PBMCs between young and old women [45].

Longevity is characterized by a balance between pro-inflammatory and anti-inflammatory agents, which act as key players. A pro-inflammatory tendency can confer high resistance against infectious diseases but, on the other hand, may increase susceptibility to inflammation-based diseases throughout life. An anti-inflammatory trend, instead, may cause an increased susceptibility to infections in pre-reproducing life and might not allow attainment of old age. The currently available knowledge is of great importance, but not sufficient to explain the secret of longevity. Data reviewed in this work are sometimes conflicting. The reason for the discrepancy is unknown, however, several factors may be involved, such as ethnic, lifestyle, cultural and genetic differences among the populations analyzed.

The studied populations are very heterogenic, the cohorts of patients present discrepancies and are not comparable. Some works were performed on healthy aged people, other works enrolled people only considering age, without considering health status and without selecting people for pathologies. Furthermore, it is known that low-grade inflammation is also associated with conditions such as obesity, diet, smoking, and physical inactivity. Therefore the balance between life style and physiological changes during aging on one hand and risk factors for age-associated diseases on the other should be taken into consideration.

However, despite all these controversial data regarding the clinical relevance of different cytokines, several studies have assigned a pivotal role in inflammatory or anti-inflammatory processes to some mediators.

According to a perspective suggested in recent years, the best candidates to become centenarians are not the strongest and most robust subjects among their age cohort, but subjects that better adapt to the environment, showing more biological plasticity. Some characteristics emerged among this group of exceptional individuals: better control of oxidative stress and remodeling of the immune system with intense anti-inflammatory activity.

Low-grade chronic inflammation is characteristic of aged people and centenarians, but long-lived are also able to avoid the main age-related diseases, thanks to the contrasting action of anti-inflammatory agents [46].

### Climate change and influence on elderly allergic diseases

There is a strong correlation between global warming and greenhouse gases and the worst estimates of the past about it were completely overcome.

The principal determinant of global warming is increasing concentration of air pollutants, as underlined by a report of World Health Organization (WHO) [47]. Last report of European Environment Agency stated that up to 96% of the European Union’s urban population is currently exposed to fine particulate matter (PM) at concentrations higher than those recommended in WHO guidelines [47]. Furthermore, increased temperatures are reported as extreme events for seasonal allergy: rising temperatures recorded in recent years cause elongation and anticipation of the pollen season, increased pollen production, spread of invasive species. Increase of temperature and solar irradiation, associated with ozone gas and particulates, determines the formation of PM, including coarse (PM_10_) and fine (PM_2.5_) [48]. In normal ambient conditions PMs are a mixture of coarse (2.5–10 μm), fine (<2.5 μm) and ultrafine particles (<0.1 μm), derived from different processes, with variable chemical matters. It should also be noted that, although ultrafine fraction accounts for less than 1% of PM mass, it represents the greatest part in terms of number of particles (typically >80%) [49]. This mixture of PM causes inflammatory effects on bronchial mucosa, increasing the risk of asthma and the number of exacerbations in subjects with bronchial hyper reactivity [50]. PMs are directly involved also in cardiovascular diseases, including vasomotor and cardiac autonomic dysfunction. Moreover, hemostatic unbalance, oxidative stress and inflammatory responses have been shown to contribute to the short-term and long-term adverse effects of pollution exposure [51]. The exposure to ozone and to fine PM (PM_2.5_) can cause adverse health effects, including premature mortality due to cardiopulmonary diseases and/or lung cancer; recent studies highlighted that mortality rates due to air pollution are different according to geographical area, suggesting region-specific air pollution control strategies [52].

#### Mechanism responsible of health effects

Pathogenetic mechanisms of PM on health effects are yet matter of controversy [53], in fact atmosphere is the medium of transit for a wide variety of biogenic particles. Among biogenic particles, the bio aerosol consists of very different types of particles, such as viruses, bacteria, mold, plant fibers or pollen, with a wide distribution in size (from nanometers to a few 100 μm). These phenomena are able to activate several inflammatory mediators in respiratory tract directly acting on respiratory mucosa and indirectly promoting increase in pollens and air pollutants concentration, with consequent effects on allergic and respiratory diseases [54].

The PM pollutants from sources such as hydrocarbons, volatile organic compounds and heavy metals can generate oxidants products. Organic compounds generate an oxidative stress through redoxing cycle of quinone-based radicals, complexing of metal resulting in electron transport and depletion of antioxidants, secondary to chemical reactions between quinones and thiol-containing compounds. Metals directly support electron transport to generate oxidants, with simultaneously decreasing of antioxidants. In addition, to direct generation of oxidants by organic and metal components, cellular responses contribute to oxidative stress: reactive oxygen species (ROS) production occurs in mitochondria, cell membranes, phagosomes and endoplasmic reticulum. Oxidative stress following PM exposure determines a series of cellular reactions that includes activation of kinase cascades and transcription factors and release of inflammatory mediators, which eventually lead to cell injury or apoptosis. Consequently, oxidative stress is a central mechanism by which PM exposure leads to injury, disease and mortality [55]. Furthermore, exposition to diesel exhaust particles and ozone in human controlled trials illustrated the important role of pollutants in immunopathogenesis of both allergic mechanisms and immune responses in airway diseases, such as asthma [56].

#### Influence of environment in elderly allergic diseases

Further findings show that some populations, especially the elderly, are particularly sensitive to short-term ozone exposure [57], with an higher risk of PM–associated hospitalization death for this population [58].

As regards the impact of pollutants on the respiratory pathologies affecting older people, some data suggest a possible role of a changed immune response as it has been demonstrated in individuals during senescence [53]. Although, atmospheric pollution plays a major role in all age groups’ health [59, 60]. For example, it is known that, in older asthmatic patients, traffic pollution exposure is the strongest predictor of poorer asthma-related QoL [61] and high daily exposure, together with obesity and not-atopic status, is associated with poorer asthma control [62]. It is also documented that mortality and risk of hospitalization in this population is increased during more intense and longer-lasting heat wave periods. Peaks of mortality and hospitalization not only coincide with the day with highest temperature of the heat wave, but also could persist for the 5 subsequent days [63]. Furthermore, in another study, cardiovascular and respiratory diseases were analyzed in an elderly population of Moscow, as they are also related to air pollution and, in generally, are considered as major causes of hospital emergency admissions [64].

Recently, many studies focus on the potential mechanisms of air pollution in inducing cardiopulmonary diseases. In fact, air pollution seems to be associated with increased plasma viscosity, abnormality of autonomic function of the heart, including increased heart rate, decreased heart variability and increased cardiac arrhythmias [65]. These findings provide possible pathways in which air pollution affects cardiovascular system. Pollutants can indeed induce oxidative processes in mitochondria, apoptosis or necrosis of macrophages and respiratory epithelial cells, resulting in decrease of host defense to respiratory infections and in increase of respiratory tract reactivity [66]. Moreover, patients with respiratory diseases, such as chronic obstructive pulmonary disease, often have a systemic defect in their antioxidant defenses and air pollution could cause significant additional oxidative stress as response to lung inflammations [67].

Nowadays health impact derived from climate change in Europe do not regard vector-borne infectious diseases. In fact, geographical areas of several vector-borne diseases and/or of their vectors are already changing in altitude, as result of global warming. Mosquito-borne parasitic and viral diseases are among those diseases most sensitive to climate. In addition, more intense weather events create advantageous conditions for infectious diseases. For example, heavy rains permit a better survival of insect breeding sites, drive rodents out from burrows and can contaminate clean water systems [68–71].

Climate changes have an impact on health due to increases in temperature, which is amplified in central parts of the cities (heat island), with a significant impact on the health of the resident population, especially for elderly and people with cardio-respiratory disease (in particular asthma and COPD). Elevated temperature reduced attendance of public areas and meeting points, such as parks or gardens. Some conditions can worsen this situation, i.e. elderly and/or subjects with chronic diseases, those with lower income, lonely subjects, immigrants and people with poor housing conditions. The reduction in hospital emergency admissions after the implementation of interventional programs supported the hypothesis of causal link between air pollution and diseases [70].

Recently, the Task Force of *Air pollution and Climate Change* of Italian Society of Allergy, Asthma and Clinical Immunology proposed a National Health Program to support the public administration of cities. In fact, it is possible to create gardens and parks with not allergic pollen plants, as suggested in different studies [71]. This aspect is important because the public gardens in our cities are visited by many children and elderly every day. In sunny and wind days this population is exposed to numerous pollens and the presence of allergen pollens.

In conclusion, the effect of global warming on health is documented in many documents, where the major warnings come from increased concentration of air pollution. In particular, urban population is currently exposed to fine PM concentrations, representing a daily problem for fragile population as elderly. Association among age, air pollution and climate changes were observed, confirming the particular interest in this population. Although, further studies are necessary to define better how air pollution and climate change are mainly responsible in elderly allergic diseases.

### Allergic conjunctivitis in the elderly

Although the eye was reported to be the first organ involved in the allergic reaction of the first described case of hay fever almost 200 years ago, ocular allergy has never received the same attention that has been given to respiratory and skin allergy [72]. On the other hand, in view of the peculiar anatomy and the prominent representation of the immune system in the ocular tissues, the eye has always represented an extremely useful model to study the immune and allergic response to environmental and endogenous stimuli [73]. There are several reasons for ocular allergy being considered the “Cinderella” of allergic diseases, despite the fact that eye involvement represents one of the major causes of poor quality of life and may often be serious enough to affect vision. At first, it is the attitude of allergists to focus mainly on respiratory and skin manifestations, considering the eye symptoms only a complication of rhinitis, under the arguable definition of rhinoconjunctivitis. Secondly, the involvement of ocular tissues in several autoimmune and systemic diseases has often been thought to be prominent competence of other medical disciplines, such as rheumatology and internal medicine. At last, the prevalent surgical commitment of many ophthalmologists has so far made difficult a proper collaboration with allergists to focus on diseases commonly considered of minor priority in both pharmacological and clinical research and practice.

The spectrum of atopic eye diseases encompasses seasonal allergic conjunctivitis (SAC), perennial allergic conjunctivitis (PAC), vernal keratoconjunctivitis (VKC), atopic keratoconjunctivitis (AKC), atopic blepharoconjunctivitis (ABC), and giant papillary conjunctivitis (GPC). Common to these diseases are a papillary conjunctivitis and, with the exception of GPC, evidence of a type I, IgE-mediated, hypersensitivity response [74]. Although each entity has unique immunopathogenic pathways, these conditions have all been described as chronic allergic conjunctivitis (CAC).

Life expectancy around the world is growing and it has been reported that by 2030 people over 65 will reach 20% of the total population [75]. In recent years, increasing attention has been dedicated to the evaluation of allergic diseases in the elderly, often underdiagnosed and confused with signs and symptoms of the physiological physical and functional deterioration with age, as well as of their impact on well-being and quality of life [3]. However, very few data are available in literature regarding the prevalence, the role and the management of allergic conjunctivitis in the aged population [76]. Most of the reported evidence, in fact, relates to contact conjunctivitis, often associated with drug use and due to impaired lacrimation in this particular population subset. However, although allergic conjunctivitis affects mainly children and young adults, an increasingly number of cases is being diagnosed in the elderly. Atopic keratoconjunctivitis seems to be the most commonly observed clinical picture and is therefore addressed in details below.

AKC was first described by Hogan in a group of highly atopic patients who developed chronic conjunctivitis, progressive corneal vascularization and scarring [77].

Currently, there is no agreement about the diagnostic criteria for AKC [78]. One proposed definition states that AKC is a chronic ocular surface non-infectious inflammatory condition, always associated with other atopic diseases (typically atopic dermatitis, but also periocular eczema, asthma, or rhinitis), occurring at any time in the course of the associated atopic disease independently from its degree of severity and with evidence of corneal involvement.

The pathogenesis of AKC involves the production of various cytokines by effector cells which are still under investigation, as their immunopathogenic roles are not fully understood. The main effector cells in AKC are mast-cells, lymphocyte T cells, eosinophils and conjunctival epithelial cells. Once activated through antigen presentation or via pro-inflammatory stimuli, they release different preformed mediators and cytokines activating a predominantly Th1 cell response (i.e. IL-2, IFNγ IL-12 and IL-8). A Th2 response is also present in these patients, but to a lesser degree. Furthermore, a peculiar feature of AKC is the increased susceptibility to infection resulting from the compromised innate immunity as demonstrated by patients’ deficiencies in keratinocyte-derived antimicrobial peptides and in both sweat and tears IgA. This results in eye infections with *Staphylococcus aureus*, as well as *Herpes simplex* virus.

The symptoms of AKC are characterized by itching, watering and a stringy mucoid discharge. Patients with AKC may have prolonged exacerbations of disease that are difficult to control and which are associated with redness, severe photophobia, extreme discomfort/pain and blurring of vision. The disease is bilateral and symmetrical. The tarsal and fornix conjunctiva are affected in all forms of the disease and are chronically inflamed. Papillary hypertrophy of both the upper and lower tarsal conjunctiva is common in the early years of the disease and giant upper tarsal papillae may occur in some patients. The conjunctiva is often so thickened by infiltrate that the tarsal vessels are obscured. Later in the course of the disease the papillae may be largely replaced by sheet scarring. Trantas dots may occur transiently at the limbus, and on the tarsal conjunctiva, during exacerbations of the disease and are usually associated with limbal swelling. Severe inflammation results in punctate corneal erosions and filamentary keratitis, which can progress to frank corneal erosions and a shield ulcer with a mucous plaque within 24 h. Plaques may result in corneal thinning, vascularization and perforation. Patients often have severe eczema of the eyelids and increased pigmentation of periorbital skin (panda eyes). There may be, at last, an associated atopic blepharoconjunctivitis.

The clinical features together with a personal or family history of atopic dermatitis are usually sufficient for the diagnosis. Laboratory investigations are needed where the diagnosis is uncertain. Diagnostic dilemmas may occur in patients who do not respond to topical therapy and in the presence of conjunctival scarring to differentiate AKC from other causes of conjunctival scarring associated with chronic conjunctivitis such as primary Sjogren’s syndrome, ocular rosacea, ocular mucous membrane pemphigoid and surface neoplasia. Relevant ophthalmic investigations include: ocular cytology, upper tarsal conjunctival punch biopsy and tear IgE assessment. Confocal microscopy may represent an additional tool for helping to refine the diagnosis when uncertain. Specific serum IgE and skin prick tests have poor value in the diagnosis.

The management of AKC is often a therapeutic challenge because of the chronicity of the disease punctuated by exacerbations of keratoconjunctivitis and complicated, especially in the elderly, by secondary infectious and herpetic keratitis, glaucoma, cataract and keratoconus [79]. The goals of treatment are to achieve symptomatic control, reduce the frequency and morbidity of corneal complications and minimize the side effects. Atopic keratoconjunctivitis therapy may be divided into conservative measures and higher risk second-line therapeutic agents [80]. Conservative measures include cold compresses, lubrication with preservative free drops, and the use of topical cromones. The addition of topical or oral antihistamines may be helpful in relieving the symptom of itch in some patients. Patients with more severe disease are refractory to these treatments and require the use of second-line topical therapeutic agents, starting with prolonged courses of topical or oral steroids to control the inflammation. The disadvantage of these agents is that they often achieve symptomatic and inflammatory control, but at the expense of steroid side-effects including raised intraocular pressure, cataracts, arterial hypertension and osteoporosis. The addition of immunosuppressive agents such as cyclosporin A and tacrolimus has been described in the treatment of AKC. Although these agents are effective and may be useful as steroid-sparing agents, they need to be prescribed carefully in view of an increased risk of local and systemic infections, particularly in aged people whose immune response may be already impaired. The use of newer biological therapies is currently being explored [81]. Agents such as infliximab (anti-TNFα), alefacept (T-cell inhibition), and rituximab (anti-CD-20) have proven to be effective in the treatment of atopic dermatitis and may have a role in patients with AKC unresponsive to conventional systemic immunosuppressive therapy. Topical or systemic antibiotics are, at last, useful for managing ulcerative blepharitis.

### Allergic asthma in the elderly

Asthma in the older adults has become a hot topic in respiratory medicine because it poses intriguing questions in terms of pathophysiological mechanisms, and challenges the traditional diagnostic approach and therapeutic algorithm that are commonly applied to younger populations. One of the main complicating factors lies in the fact that the respiratory system is particularly susceptible to time-related deterioration in structure and function [82], approaching a condition that is often confused with that of chronic obstructive bronchitis or emphysema. The prevalence of asthma in older populations is not different from that of younger age groups; however, geriatric asthma presents with clinical and functional features that make its management a potential complex and complicated task; among them, the atopic condition, or more precisely, the lack of it. The question is not whether asthma in the elderly is a rare disease, but rather whether *allergic* asthma is a rare entity in the elderly. The immune system undergoes an involution process with increasing age; as a consequence the immunoglobulin production, including IgE, declines leading to the misconception that asthma in the elderly has a non-allergic pathogenesis. The clinical implications of this phenomenon are that atopy is relegated to a corner in the assessment of the geriatric respiratory patient. This has greatly contributed to underdiagnosis and misdiagnosis of asthma in the most advanced ages.

Atopy refers to the predisposition to develop allergic diseases, such as allergic asthma, and describes the ability to mount IgE responses to common allergens. The initial observations on general population samples of a reduction of atopy with age reinforced the theory that *allergic* asthma is a rare nosologic entity in the elderly. With the aim of providing a definite answer to this doubts, Scichilone and colleagues [83] conducted a systematic revision of published articles on the topic, and found that, overall, the prevalence of allergic sensitization is lower in the most advanced ages. These findings were confirmed by a survey conducted on large European population [84], which showed that positive skin prick test for all allergens tended to be less common in older adults than in younger ones. It must be stated, however, that the published literature in the field suffers from the lack of longitudinal studies to confirm the reduction in the atopic condition with ages. In other words, a lower prevalence of atopy in the elderly as demonstrated by cross-sectional observational studies could simply reflect a lower prevalence of atopy in the same individuals at younger ages. Another factor to be kept in mind is that, when defining atopy, increased total IgE levels and positive skin prick test to allergens are used interchangeably, whilst the use of specific IgE towards aeroallergens could be more appropriate. A distinction should be made between asthma occurring for the first time in older ages (late-onset asthma), and asthma that begins in younger ages (early onset asthma). The former is historically believed to have a lower likelihood of allergic features [85]. The assessment of the features of late onset asthma is complicated by the fact that some investigations included as “late” asthma cases presented in adulthood. In addition, the so-called “recall bias” could have affected the definition of asthma in the elderly as late-onset rather than long standing.

Persistent antigen exposure characterizes the natural course of allergy: the continuous stimulation with the same antigenic proteins leads to the expression of new surface molecules together with the expansion of effector and memory cells. This translates into a decrease in the number of naive cells and an increase in the number of sensitized cells. Therefore, in allergic asthma the naive T cells tend to decrease whereas the T lymphocytes are constantly stimulated by the aeroallergens. Of note, age also affects mast cell and basophil mediator releaser and the responsiveness of other inflammatory cells to the chemoattractive and activating signals of mast cell and basophil mediators, which influence the inflammatory cascade that follows the IgE receptor bridging.

The haematopoietic compartment of bone marrow is replaced by fatty adipose tissue with increasing age [86], with a functional decline in stem cell precursors which, however, seems to occur only under stressing conditions; in basal conditions the absolute numbers of eosinophils and basophils in young versus older subjects does not differ [87]. An old investigation carried out in elderly subjects showed that paracortical and medullary zones of lymph nodes were strongly reduced, with lower germinal centres [88]. Furthermore, aging is associated with changes in the function of lymphocytes, such as a lower antibody response, a decreased ability to produce high affinity antibodies and reduced IgG isotype class switching [89]. Increasing age is also associated with an attenuation of dendritic cell function. Agrawal et al. [90] reported that peripheral blood dendritic cells are similar in young and aged subjects, but migration and phagocytosis are impaired in older adults. Aging is characterized by macrophage dysfunctions consisting in the reduction of expression of TLRs, secretion of cytokines following activation, and phagocytic ability [91].

Scichilone and colleagues [83] point out that, despite lower prevalence of atopy in the elderly, the prevalence of the allergic component remains high in older individuals suffering from asthma, and this may have clinical consequences. For example, the age-related reduction in the production of IgE does not decrease the efficacy of a pharmacological approach with anti-IgE in the severe forms of asthma occuring in older ages. Although less frequent, the allergic reactions in older adults can be even worse than in younger people, because of the inability of other organs and systems to compensate. For example, a systemic anaphylactic reaction may have dramatic consequences in elderly subjects in whom the cardiovascular system is not able to effectively compensate. In addition, atopy can interact with other conditions (primarily viral infections), eliciting more severe exacerbations in the elderly. Regardless of age, the assessment of atopy is crucial in the comprehensive evaluation of the chronic respiratory patient, in that, it provides the unique opportunity to act on the environment (removal of allergens) and to modify the natural history of the allergic condition with allergen immunotherapy. Indeed, age per se does not preclude the use of allergen desensitisation.

The relationship between the presence of the allergic component and the geriatric age may be complicated by the incorrect interpretation of skin testing. This may be due to the age-associated reduction in skin reactivity of the elderly to histamine and allergens. In addition, the area of the skin where the test is performed could be atrophic with decreased cellularity, thus influencing the response to allergens. The reduction in blood vessels and mast cells reduces the binding sites for allergen and the release of histamine to produce wheal and flare. Finally, mast cells function can be impaired by prolonged exposure to sun, as often occurs in the elderly. A negative skin prick test does not rule out completely the presence of an allergic condition. The concept of localized mucosal allergy with the production of specific IgE antibodies in the absence of atopy has been put forward, and is currently an object of investigation. Taken together, these observations allow to speculate that the immunological and inflammatory responses to stimuli may be different in the older ages, implying a different history of the disease. It is logical then to hypothesize that asthma in the elderly will have to be treated accordingly.

### Allergen immunotherapy in the elderly

#### General aspects of AIT

Allergen (specific) immunotherapy (AIT) was introduced in clinical practice more than one century ago, with the supposed aim of “vaccinating” against hypothetical “aerogenic toxins”. Despite the rationale was wrong, the procedure resulted to be clinically effective and rapidly spread. Subcutaneous injections (SCIT) remained the only mode of administration for more than 70 years, when new modalities were proposed, with the aim of improving the safety and convenience. Among the various routes proposed, the sublingual one (SLIT) rapidly gained credibility, so that it was accepted as a viable alternative to SCIT in all official documents and guidelines [92]. In general, the clinical efficacy of SLIT and SCIT are equivalent, although SLIT has a more favourable safety profile [93].

To date, the practice of SCIT is sufficiently standardized, as testified by position papers and practice parameters [94–97]. On the other hand, SLIT can be administered as drops, monodose vials or tablets, with variable timings and doses depending on the manufacturer. In the last decade, highly standardized products in tablets (grass, mite, ragweed) have been approved as drugs by EMA and FDA. The aim of AIT is of interfering with the immune response to the offending allergen, thus inducing a tolerance that results in a reduction of symptoms and medication intake upon natural exposure to the allergen itself. SCIT usually consists of an updosing phase (with gradually increasing doses of the allergen) followed by a maintenance phase, were the maximum dose is given at regular intervals (usually monthly) for 3–5 years. With SLIT, due to the favorable safety profile, the updosing phase is absent or very short, and the maintenance is given on daily basis.

#### Mechanisms of AIT

Differently from traditional medications, AIT is not a mediator or receptor inhibitor but rather a biological response modifier. Its mechanisms of action are multiple and complex, and exerted at various levels of the allergen-induced inflammatory reaction. The complexity of the immune-mediated actions of AIT have been extensively described in many reviews [98–100].

Briefly, the sensitizing molecules contained in the allergen extract (independent of the mode of administration) are recognized by antigen presenting cells, processed and presented to Th lymphocytes. This results in the activation of T regulatory (Treg) cells which, through the production of numerous cytokines (IFN-γ, IL-10, IL-12) induce:

- A relative shift from the Th2 towards the Th1 phenotype (allergic subjects have an imbalance in the Th2/Th1 lymphocyte in favour of Th2). This reduces the production of IL-5, IL-4, IL-13 that specifically favour the allergic inflammation.
- A decrease in allergen-specific IgE synthesis.
- An increased production of allergen-specific IgG4, which block the IgE-facilitated antigen presentation.

All these modification are not permanent, but they are long-lasting, as testified by the fact that the clinical benefit obtained in a successful course of AIT may persist for years after discontinuation. In addition, the disease-modifying effect can affect the natural history of allergic diseases (for instance reducing the risk of asthma onset) [101].

#### The “problem” of AIT in the elderly

As a matter of fact, the use of AIT in elderly people has been until the last decade a neglected aspect. Looking at the available literature, and particularly at randomized trials, it appears that patients aged 65 years or older are never included in the study population [94–96]. Guidelines are usually elusive. Some of them indicate older ages as a relative contraindication to AIT [102], some others do not mention this aspect [97], and in others caution is recommended, especially concerning the problem of safety and the treatment of severe reactions [103]. This fact is the consequence of two consolidate beliefs. First, allergic diseases are expected to be relatively rare in aged patients and, second, it is thought that with ageing the immune system loses its ability to efficiently respond with specific IgE. On this background, AIT has never been considered a therapeutic option for older people. The context is further complicated by the frequent presence of comorbidities, and consequent poly-pharmacotherapy, that can interfere with AIT (e.g. ACE inhibitors, betablockers or major antidepressants).

Indeed, in the recent years it has become clear that the allergic respiratory diseases are not a rare situation in elderly people [104]. In fact, the prevalence of respiratory allergy (asthma and/or rhinitis) have been shown to range between 5 and 10% in various studies [105, 106]. Lombardi et al. [107] have recently shown that the prevalence of allergic sensitizations with clinical manifestations in older subjects approximates 50%. In another cross-sectional study, involving about 2000 elderly patients with a clinical suspicion of allergy, the prevalence of asthma, seasonal rhinitis and perennial rhinitis were found to be 5, 13 and 17%, respectively [108]. Thus, despite the problem is considered of scarce relevance, the available data testify for a not negligible social impact. Concerning the immune system, the phenomenon of “immune-senescence” is well known since many years. It is characterized by an overall reduction in: bone marrow proliferation, antigen-presenting function, capacity of mounting an antigen-specific humoral response [89, 109, 110]. This would result in a decreased capacity of synthesizing IgE. It is true that in several studies, the acquired immunity seem to decline with age [for review see 84]. On the other hand, several studies have demonstrated that the Th2-driven response in the elderly does not differ from that of younger subjects [111, 112] and that the production of IgE does not always decline [113]. In addition, the nasal cytology aspect in elderly subjects with allergic rhinitis is identical to that of young patients [114], and a local nasal specific IgE response is detectable also in aged patients [115].

#### The evidence

Due to the above mentioned limits, it is easy to understand that there are very few clinical trials exploring the effects of AIT in elderly subjects. In a nonrandomized open controlled study of SCIT for birch and ragweed allergy involving a total of more than 100 patients, the clinical benefit was identical in patients aged less or more than 55 years, and greater than in controls not receiving SCIT [116]. Subsequently, other Authors [117] performed a retrospective study where the effects of SLIT (symptom/medication scores, bronchial hyperreactivity, pulmonary function) were compared between young adults (18–55 years, n = 49) and older patients (55–65 years, n = 40). They found no difference between the two age groups in the magnitude of the achieved clinical efficacy. More recently, Bozek et al. [118] explored the effects of SLIT in 108 patients aged 60–75 years and receiving dust mite SLIT or placebo for 3 years. Main findings were that total nasal scores decreased by 44% VS baseline in the SLIT and by 6% in the placebo group and medication score decreased vs baseline 35% in SLIT group, although no change was observed between groups at the specific nasal challenge. The same Authors assessed the efficacy of pre-seasonal grass pollen AIT in subjects aged 65 years or more and suffering from seasonal rhinitis [119]. Sixty patients were randomized to receive AIT or placebo for 3 years, and the area under the curve for symptoms + medications score was evaluated at the 3rd year. The reduction of this parameter versus baseline was significantly greater in the active group, and the symptom score, medication score and combined decreased by 50% on average.

Polytherapy may represent a problem in selected cases and with selected drugs that in principle can interfere with the course and treatment of severe reaction if any occur. For instance, beta blockers may counteract and monoamine oxidase inhibitors may potentiate the effects of epinephrine, when it is given for anaphylactic reactions (one of the possible although rare side effects of AIT). Indeed, the new selective betablockers do not carry this risk, and in fact have been indicated only as relative contraindication in the more recent literature reviews, whereas monoamine oxidase inhibitors do not represent a contraindication. The same holds true for ACE inhibitors, for which the risk of worsening hypotension in anaphylaxis has not been substantiated [97, 120]. Cardiovascular diseases are not per se a contraindication to AIT as well. Venom AIT represent a special case, since the risk of anaphylaxis after sting in sensitized subjects (especially the elderly) is always greater than any possible risk due to cardiovascular diseases or drugs interfering with epinephrine.

In conclusion, there is increasing evidence that allergic disorders in the elderly are not rare, and an efficient IgE response can be expected also in this age group. On the other hand, the few evidence so far available for AIT, consistently confirm that the treatment is effective also in older subjects. Thus, provided that the IgE-mediated nature of the disease is clearly ascertained and that the offending allergen is unequivocally identified, AIT can be prescribed also in the elderly, with the same indications and modalities applied in young adults. In the elderly, additional care should be paid in assessing the presence of comorbidities, the use of multiple drugs, and the expected compliance.

### Chronic urticaria in the elderly

As all organs, the skin ages with structural and functional consequences that may lead to clinical conditions. The “skin aging” hallmarks are: (1) atrophy of the epidermis and dermis due to loss of hydration; (2) progressive loss of function and structural integrity resulting in impaired immune response and skin barrier function; (3) skin vascular impairment; (4) metabolic imbalance of reactive oxygen species, and components of the extracellular matrix [121]. Aged skin is characterized by atrophy, wrinkling, fragility, alterations in pigmentation, a higher frequency of benign and malignant tumours, and a greater tendency to xerosis [122]. These factors contribute to greater susceptibility to dermatologic diseases in individuals over the age of 65 years. In this context, one of the most common skin diseases is the allergic contact dermatitis (ACD), which is mainly due to the direct exposition to nickel sulphate (11–12%) and fragrance/balsam of Peru (7–9%). Atopic dermatitis (AD) is much less common in the elderly in comparison with children and young adults. AD is associated with seasonal mucosal allergies, asthma and positive prick tests to various allergens [123]. Late onset AD, without the usual history of atopy, may explain eczema of unknown origin and negative patch tests. Scabies should enter the differential diagnosis in generalized dermatitis, as institutional acquisition of scabies is common in the elderly.

Chronic urticaria (CU) is a common nosological entity in older individuals. This is especially true for the spontaneous form of the disease, although there are few data regarding epidemiology and clinical features. Urticaria is defined as itchy wheals with or without angioedema that usually persists for less than 24 h. According to current EAACI/GA2LEN/EDF/WAO guidelines, CU can be classified as spontaneous, physical, or other [124]. Chronic spontaneous urticaria (CSU) is the most common subtype of all forms of non-acute urticaria, and is characterized by wheals that develop independently of external stimuli and last for a minimum of 6 weeks. In general, the underlying causes of CSU are difficult to identify in most patients. It is estimated that about 0.5–1% of the population suffers from CSU, and that about one quarter of the population has experienced urticaria at some point during their lives. Both sexes can be affected, but in general, females appear to suffer from urticaria nearly twice as frequently as males.

The pathogenic mechanisms of CSU are unclear. Evidence of an autoimmune etiology is reported in about 45% of CSU patients, remaining unknown in the remaining subjects. Circulating autoantibodies specific to high-affinity immunoglobulin E (IgE) receptors or dermal mast cell bound IgE activate mast cells and induce degranulation with cytokine release [125]. The autologous serum skin test (ASST) is a screening test for autoreactivity; if positive, it suggests the presence of circulating histamine-releasing factors of any type, and not only of functional autoantibodies. Atopy is proposed to play some role in the pathogenesis of CSU, especially in the aspirin-intolerant CU phenotype. Several hypotheses exist regarding the relationship of atopy and aspirin intolerance; however, the exact association remains unclear.

Systemic diseases that may induce elderly urticaria should be carefully evaluated. Findings derived from the Mayo Clinic’s electronic database revealed that patients presenting a new diagnosis of CU at older ages were more likely to have underlying monoclonal gammopathy of undetermined significance (MGUS). Some authors have also emphasized the role of drug-induced urticaria in the geriatric population [126].

Angioedema in the absence of urticaria can be due to overproduction of bradykinin [127]. It is exceptional that hereditary deficiency in the C1 inhibitor (HAE-C1-INH) has its onset in the elderly. It is more frequent that acquired C1 inhibitor deficiency (AAE-C1-INH) presents in the older age, and it is characterized by activation of the classical complement pathway and accelerated catabolism of C1-INH due to lymphatic tissue neoplasms or autoimmune diseases. The prevalence of angiotensin-converting enzyme inhibitor angioedema (AE-ACEi) is relatively high, ranging 0.1–2.2%, and it should be suspected in all patients with AE who are receiving ACEi. Normal levels of complement factors help to reinforce the clinical suspicion and to rule out the possibility of AE with C1-INH deficiency.

In a retrospective investigation conducted on a large cohort of patients with CU from the National Health Insurance Research Database of Taiwan, Chen and colleagues found that a quarter was in the age range of 60–79 years and 3.4% were 80 years and older [128]. A study performed by Magen and collaborators on 1598 adults suffering from CSU found that 9.4% were elderly [129]. Interestingly, the latter showed lower rates of angioedema and dermographism, as well as fewer wheals and ASST positivity. At variance with the younger populations, no sex differences were demonstrated in the elderly, whereas comorbid conditions, such as diabetes mellitus, chronic renal failure, hypertension, Hashimoto’s thyroiditis, and malignancies were more prevalent in the elderly [129].

Ventura et al. [130] found that CSU due to infections was more common in the elderly, especially the CSU forms related to Helicobacter pylori and those related to the sensitization to the parasitic nematode Anisakis simplex. Ban et al. [131] reported that in the elderly with CU there is a high prevalence of AD compared with younger groups. The prevalence of AD was equal to 37.8 versus 21.7%, respectively (p = 0.022). Disorders of the skin barrier are found in subjects suffering from AD and this leads to the colonization of Staphylococcus aureus, the degraded ceramide of which penetrates the skin barrier and induces IgE sensitization.

#### Elderly CSU diagnosis

As a general concept, the diagnostic approach to CSU is not influenced by age. Indeed, the international guidelines do not recommend a specific work-out for elderly populations, although a careful assessment of the drug history should be encouraged (especially for aspirin and ACE inhibitors), and the presence of autoimmune or neoplastic diseases ruled out [132]. An extended laboratory and instrumental tests should be reserved to specific conditions as judged by the physician.

#### Elderly CSU treatment

The avoidance of causal and/or triggering factors is the first choice in the management of CSU. This is however a rather complicated task, given that in the majority of cases the recognition of these factors is unsuccessful. The pharmacological treatment of CSU raises a safety issues mainly due to concomitant diseases and polypharmacotherapy, which are common in older subjects and may cause drug-to-disease and drug-to-drug interactions. Indeed, the potential for drug interaction increases with age and with the number of drugs prescribed [133]. According to the international guidelines, the management of CSU is based on a step-wise approach, with sequential steps to be implemented according to the clinical response. The mainstay treatment for CSU in the elderly consists of new-generation non-sedating antihistamines. First generation H1 receptor antagonists are lipophilic and therefore may cross the blood–brain barrier. For this reason, elderly individuals may be at increased risk of adverse effects involving the CNS (confusion, sedation, dizziness, sleepiness, and impaired cognitive function). Agostini et al. [134] showed that the administration of diphenhydramine in elderly hospitalized patients was associated with increased risk of cognitive decline compared with non-exposed patients. Because of the lack of specificity for the H1 receptor, due to activation of dopaminergic, serotoninergic, muscarinic and cholinergic receptors, the first-generation antihistamines have also additional adverse effects with an higher risk of urinary retention, constipation, as well as arrhythmias, peripheral vasodilatation, and postural hypotension. First-generation antihistamines should therefore be prescribed with extreme caution in elderly patients. Second-generation oral H1 anti-histamines have a safety profile with reduced capacity to induce CNS-related adverse effects as they have a low potential to cross the blood–brain barrier, and provide selective H1 receptor blockade without anticholinergic activity. It is instead important to remember that second-generation oral H1 antihistamines potentially requiring a dose reduction in patients with hepatic diseases or dysfunction include cetirizine, ebastine, levocetirizine, and loratadine. Those potentially requiring a dose reduction in patients with renal dysfunction include cetirizine, ebastine, fexofenadine and levocetirizine. Conversely, no dosage adjustment for desloratadine is required in the elderly healthy subjects [135]. Pharmacokinetic/pharmacodynamic studies in special populations indicate that bilastine’s dose adjustment is not necessary in elderly patients, or in hepatic or renal insufficiency [136]. The safety of second-generation oral H1 receptor antagonists up-dosing has not been systematically evaluated in the older population.

With regard to the third-line therapy for CSU, very few studies explored the effect of montelukast in the elderly. Available data reassure on the safety profile of montelukast in the elderly patients, and dose adjustments are not necessary even in individuals affected by renal or hepatic failure. It must be stated, however, that montelukast can interact with drugs that interfer with the CYP3A4, CYP2C8, or CYP2C9 systems.

Alternative treatment options have not been systematically evaluated in elderly patients with CSU. Corticosteroids and cyclosporine could be used with caution in cases resistant to therapy. Old age may be a risk factor for particular corticosteroid-associated side effects and adverse reactions (diabetes, cataract, and osteoporosis).

The anti-IgE humanized monoclonal antibody (omalizumab) has been successfully tested in subjects with CSU up to 75 years of age, namely 4–9% in the study of Maurer and colleagues [137], and 3.7–5.2% in that of Saini and colleagues [138]. Kaplan and coauthors showed that anti-IgE treatment was effective irrespective of age [139]. No omalizumab dose adjustment is recommended for elderly patients or those with impaired renal or hepatic function, and there is no evidence of clinically significant drug interactions [140]. The literature contains sporadic reports of older patients with difficult-to-treat CSU and/or chronic inducible urticaria treated with omalizumab; in one case the highest age was 82 years. Nowadays, specific data are not available on omalizumab treatment in elderly patients with CSU.

In conclusion, CSU in the elderly induce severe disability and decrease in quality of life and CSU can be associated with other diseases (autoimmune, infectious, neoplastic, etc.). Polypharmacological treatment is very frequent in the elderly patients, and should be taken into account in the treatment of CSU, as it can induce drug interaction due to the various comorbidities and changes in pharmacokinetics and pharmacodynamics. Like adults and children, the mainstay treatment for CSU in the elderly consists of new-generation non-sedating antihistamines.

### Food allergy in the elderly

The incidence of IgE-mediated allergic reactions is rising worldwide. The changes of the demographic parameters let foresee such a meaningful increase in the proportion of allergic patients in the next few years, so that the possibility of missed recognition or under diagnosis should be avoided [141]. In particular, a recent paper on food allergy carried out in a geriatric nursing home on subjects with a mean age of 77 years showed that 40% of the patients had specific IgE to respiratory allergens and 24.8% to food allergens, with a direct correlation to the positivity of skin prick tests (SPT). According to the authors the problem of food allergy (FA) in the elderly is underestimated, because epidemiological studies are focused mainly on children and on adults, as if the elderly could not be affected by these allergies [142].

An incidence of 5% FA in people over 65 was diagnosed in outpatients of the Allergy Section at Hospital Vall d’Hebron, in comparison to the 36% diagnosed in people aged between 40 and 65 and to the 6 9% in the youngest [75]. The profile-sensitization was not much different among the groups, except for the *Rosaceae* fruits and fish, which were found to be much more frequent in younger people [75]. In addition, data from the literature show that with regard to the clinical manifestations anaphylaxis from food allergens is less frequent in the elderly in comparison with young subjects [143]. Another recent work has shown that in a geriatric Immunoallergology Unit one-third of the patients over 65 who were afferent to the structure and had been visited and subjected to specific allergy tests for suspected food allergy were positive to SPT to food; it confirmed the diagnosis of FA [76].

To understand the reasons of FA occurrence in the elderly it is appropriate to reevaluate the physiological changes that occur in the mucosal immune system. In fact it should not be forgotten that the gastrointestinal tract is the largest immunologic system with an important number of lymphocytes; part of them are isolated and part of them are aggregated in lymphatic structures called Peyer’s patches. In addition, this system plays a key role in all reactions involving mucosal immunity, including FA. It takes particular importance in the elderly due to the physiological involution of the thymic gland and its related functions [144].

As a matter of fact it is in the intestinal mucosa and in the immune related tissue, the so called gut associated lymphoid tissue, that occur the modifications which are responsible for the change in the oral tolerance. Generally the oral tolerance that is established at a young age is maintained even over 65, unless new antigens which are unknown to the immune system [145] are introduced. As a consequence, the induction of tolerance to new antigens is reduced in elderly mice. These data show clearly that the answers of the Secretory Antigen-specific IgA (S-IgA) and oral tolerance is modified in aged mice; even with regard to the protection from infectious diseases, some alterations that should be better investigated can occur especially in humans [146]. Another important factor responsible for changes in oral tolerance is the alterations of the gastrointestinal permeability. The intestinal barrier integrity interruptions may arise after the appearance of alterations in the gastroenteric mucosa [147]. They occur mainly at the level of tight junction (Tj) for alterations of both canonical and non-canonical activation of NK-κB pathways in intestinal epithelial cells [148]. These structures, that generally provide a “barrier” effect, become more permeable both for the alteration of mucosal epithelium due to inflammatory diseases of the gastrointestinal tract and for the effect of some cytokines whose secretion is increased in the elderly, such as IL-6, TNFα and IL-β, responsible for a “pro-inflammatory” status, the so called “inflamm-aging” [13, 149]. In particular the epithelial cells, which are responsible for the production of large amounts of cytokines, including the IL-β, increase the permeability of the mucous membrane through the reduction of the proteins of the Tj and occludens zonula [150]. This weakening of the “barrier effect” results in a modification of the mechanisms of oral tolerance and thus predisposes to FA. Another key factor is the presence of inflammatory cytokines at this level. A recent study confirmed it proving that in the biopsies of the intestinal mucosa of old baboons there is an up-regulation of micro RNA, miR-29a and of inflammatory cytokines IFN-γ, IL-6 and IL-1β[1511]. The role of S-IgA should not be forgotten too. These immunoglobulins play a critical function both for the maintenance of the intestinal microbiota and consequently for the modulation of local immune responses [151].

With regard to the IgA there are conflicting studies about the maintenance of an optimal level of IgA in the gastroenteric tract in the elderly. Although the IgA reduced levels can rather reflect an alteration of the migration of plasma cells secreting IgA from effector to inductor sites, rather than their actual numerical reduction [152]. However it is reported that Antigen-specific responses of IgA type are weaker in elderly animals. In addition, young mice have less somatic mutations of IgA if compared to older mice, and the analysis of more than a million VH sequences showed that the IgA repertoire of the young differs from that of the elderly [153]. It is not yet fully clarified how these differences can result in the decreased efficacy of the security role exerted by the IgA in humans.

Moreover, the habit of eating raw fish, common in Italy and in other countries, is connected to the possibility of developing allergic sensitization to *Anisakis simplex*, whose incidence increases over the years because of the increase of exposure [154]. This sensitization is expressed with typical clinical manifestations of FA including especially hives, but also more severe reactions such as asthma and anaphylactic shock [155]. In particular it has been shown that even in this case a situation of impaired gastric permeability, which can promote massive penetration of allergens and induce important clinical manifestations, can also coexist [156].

According to Diesner et al. [142] during senescence the changes that occur in the innate immune system, as well as in adaptive, justify the appearance of FA. At the same time the lack of some micronutrients, including zinc and iron or vitamin D may be a risk factor. In particular the reduced level of zinc, frequent in elderly subjects, is responsible for the reduction of Th1 cytokines, while it does not interfere with the production of Th2 cytokines, that promote the increase of allergic diseases in the elderly [157]. The reduction of iron, which occurs often in the elderly, is another risk factor. In fact, it is responsible for a diminished antibody response, especially with regard to the IgG4 subclass that can prevent activation of effector cells through the capture of allergens before they can act the IgE-mediated binding [158].

Even vitamin D could play a role through T cells and antigen presenting cells by promoting tolerance via inhibition of inflammatory responses and the induction of regulatory T cells [159]. Another contributing factor is represented by atrophic gastritis, which is a frequent disease among the elderly, or from the excessive use of alcohol, proton pump inhibitors or antacids. As a consequence undigested proteins that take on allergenic properties would remain in the stomach triggering FA [160].

In conclusion, FA represents an emerging problem in the elderly, complicated by gastrointestinal mucosa permeability and impaired function of the local immune system.

### Drug allergy in the elderly

We define “adverse drug reactions” (ADRs) any untoward reactions to a medication. ADRs may be broadly divided into two types: A (reactions occurring in most normal patients, given sufficient dose and duration of therapy: common and predictable) and B (drug hypersensitivity reactions restricted to a small subset of the general population: rare and unpredictable). A drug allergy (DA), i.e. an allergic drug reaction, is an adverse drug reaction that results from a specific immunologic response to a medication, therefore a Type B reaction (10–15% of ADRs). It is a reaction which cannot be usually predicted and occurs in a susceptible kind of patients. There are exceptions: predictable hypersensitivity reactions to abacavir, dapsone, carbamazepine, allopurinol end flucloxacillina in patients with certain leucocyte antigen (HLA). Signs and symptoms are different from the pharmacologic actions or the collateral effects of the drug. DA account for about 6–10% of all ADRs, but for up to 10% of the fatal reactions [161]. The term “immunologic drug reaction” is also used to describe these reactions. Immunologic drug reactions can be mediated either by humoral immunity (IgE, IgG), or by lymphocytes. There are different systems for classifying DA. One is based upon the immunologic mechanism and identifies four categories (Gell and Coombs): Type I, immediate in onset and mediated by IgE and mast cells and/or basophils, Type II, delayed in onset and caused by antibody (usually IgG)-mediated cell destruction, Type III, delayed in onset and caused by IgG-drug immune complex deposition and complement activation and Type IV, delayed in onset and T-cell mediated (Type IV reactions may be further subdivided into IVa, IVb, IVc, and IVd). Types I and IV reactions are far more common than types II and III, the latter usually following prolonged, higher dose therapy. Most medications cause just one type, although certain drugs, such as penicillin, or glucocorticoids can induce all types [162]. A second classification is based upon the timing of symptom onset. The WAO has recommended that, based upon the timing of the appearance of symptoms, immunologic drug reactions be divided into immediate reactions (onset within 1 h after exposure) and delayed reactions (onset after 1 h) [163]. However some IgE-mediated reactions may appear after 1 h, particularly if the drug is assumed per os and especially if together with food. Usually, delayed reactions begin after weeks of continuous treatment. The reaction, also called “drug-induced hypersensitivity syndrome” (DiHS), is characterized by fever, rash, and multi organ involvement, and may or may not be associated with eosinophilia and lymphocytosis. It can persist for weeks to months, even after the medication is stopped. One such disorder is “drug rash with eosinophilia and systemic symptoms” (DRESS), a systemic drug reaction that begins 1–12 weeks into continuous treatment [164]. The liver (hepatitis) and heart (hypersensitivity myocarditis) may be affected.

While the prevalence and the risk factors associated with the ADRs in the general adult population have been well documented, much less is known about the ADRs, and in particular about the DAs, in the elderly population.

A number of factors in older individuals contribute to their increased risk for developing a drug-related problem. These include female sex, frailty, coexisting medical problems, memory issues, and use of multiple, interacting, prescribed and non-prescribed medications [165].

A study of Alhawassi et al. [166] review the Literature to estimate the prevalence of the ADRs in the elderly in the acute care setting and to identify the factors associated with the increased risk in the elderly. The mean prevalence of the ADRs in the elderly in the studies included in this review was 11.0% (95% confidence interval [CI] 5.1–16.8%). The median prevalence of the ADRs leading to hospitalization was 10.0% (95% CI 7.2–12.8%), while the prevalence of the ADRs occurring during hospitalization was 11.5% (95% CI 0–27.7%). There was wide variation in the overall ADRs prevalence, from 5.8 to 46.3%. The Authors conclude that the ADRs constitute a significant health issue for the elderly in the acute care setting. While there was wide variation in the prevalence of ADRs in the elderly, according to this study about one every ten admissions of elderly patients is due to ADRs and 10% elderly patients will suffer from an ADR during the hospital stay. Older female patients and those with multiple comorbidities and medications appear to be at the highest risk of ADR in the acute care setting.

Drug-related hospitalizations account for 2.4–6.5% of all medical admissions in the general population; the proportion is much higher for older patients [161, 167]. In the United States, it is estimated that annually from 2007 to 2009 there were 99,628 emergency hospitalizations for adverse drug events in individuals aged 65 years or more, and two-thirds of these were due to unintentional overdoses [168]. A meta-analysis found a fourfold increase of the rate of hospitalizations related to adverse drug events in older adults compared with younger adults (16.6 versus 4.1%); it was estimated that 88% of the ADR hospitalizations among older adults, and 24% among younger adults, were preventable [169].

A meta-analysis on UK hospitals found that the frequency of drug-related hospitalizations was between 2.4 and 6.2% [170] and many of these were considered preventable [171]. A recent study in the Netherlands [172] that about 41,000 hospital admissions per year were related to ADRs and about the half were potentially preventable. The risk was doubled if patients were 65 or older. The elderly are particularly at risk for drug-related problems [173]. About 30% of hospital admissions of patients aged 65 and over are due to ADRs, most of which are preventable [174]. It is also of concern that the likelihood of severe long-term outcomes such as disability and death following ADRs is significantly higher in the elderly population [175]. Epidemiological studies have found that the classes of drugs most commonly associated with ADRs in the elderly include diuretics, warfarin, non-steroidal anti-inflammatory drugs (NSAIDs), selective serotonin reuptake inhibitors, beta-blockers and angiotensin converting enzyme (ACE)-inhibitors, chemotherapy for cancer, glucocorticoids [176–180].

The data of the Italian Group of Pharmacovigilance in the Elderly (GIFA) have shown that age is not an independent risk factor but it is associated with significant changes in pharmacokinetics and pharmacodynamics that predispose the elderly patients to higher risks. Polypharmacy, changes in pharmacokinetics/pharmacodynamics and drug interactions are the main factors predisposing the elederly patients to ADRs [181].

Previous data demonstrates that most of the ADRs in the elderly are “preventable”, therefore they are not Type B reactions. Ventura et al. [182] investigated the prevalence of unpredictable drug adverse reactions among patients admitted to the Emergency Departments (EDs) of three large Italian hospitals in the period 2005–2008. Clinical characteristics and demographics were carefully recorded in a dedicated database. The assessment of the drug reactions was carried out by an allergist after the first emergency evaluation. Over the considered period, 56,031 patients were admitted at the ED, 2644 (21.2%) of which for ADR. Out of those patients, 55 (2.1%) were identified as unpredictable ADRs. In 96% of the cases the clinical presentation was cutaneous and antibiotics were the most frequently responsible drugs. Patients over 65 years accounted for 37% of the reactions. In those patients the multiple drug regimens were significantly more frequent, as well as the presence of comorbidities.

In 2007 the Finnish National Agency for Medicines made available on its website (http://www.nam.fi/publications/tabu) the summary of reports received between 1973 and 2006 about ADRs (including vaccines) in patients ≥75 years. Approximately one-third of the 2013 cases concerned anti-infective drugs. The reactions described had the characteristics of type B reactions.

Therefore it can be concluded that: (I) today there are no adequate studies that define the epidemiology of DA in the elderly and (II) most of the described ADRs are not characteristic DAs. By using the evidence from the studies it is only possible to estimate that the DAs in the elderly have a prevalence of 0.6–2.1% but constitute as much as 10% the fatal adverse reactions.

There are three certainties about the DAs in the elderly: (a) giving the rising age of the population (a risk condition) it can be concluded that at the present time is absolutely essential to proceed with adequate epidemiological studies; (b) in the elderly the diagnosis must be made according to current guidelines [183–185] and there should therefore be a “systems” approach, which has a historic equivalent in the geriatric “multi-dimensional” approach. Evaluation involves a meticulous history of past and present ADRs, the acquisition of additional information from medical records and an analysis of the temporal patterns between drug administration and onset of symptoms. Once this data has been assembled, it must be combined with our knowledge about the specific types of allergic reactions caused by the various classes of drugs, in order to identify the potential culprit agents. Advances in drug allergy include identification of HLA associations for penicillin allergy and a micro RNA biomarker/mechanism for toxic epidermal necrolysis [186]. Finally (c) the correct choice of therapy in the elderly population may be the administration of an unrelated medication meeting the criteria of a “lean prescription” and Beers’s criteria [187] a careful administration of a related medication or the desensitization to culprit drug.

### Anaphylaxis in the elderly

Anaphylaxis is a severe and potentially fatal hypersensitivity reaction that can affect patients of all ages [188]. The elderly differ from other age groups in terms of risk factors because significant comorbid conditions, such as cardiovascular or cerebrovascular disease, hypertension and cardiac arrhythmias may occur. Moreover, in this population of patients compensatory mechanisms can be physiologically reduced or decreased due to the intake of antihypertensive drugs like β-blockers and ACE inhibitors. Also, trigger agents are usually different than younger patients. All these factors influence the outcome and the response to treatment during anaphylaxis in the elderly. Therefore elderly patients must be considered particularly vulnerable to severe anaphylaxis [189].

Campbell et al. [143] studied presentation and management of patients with anaphylaxis who were 50 or 65 years or older in a consecutive cohort of patients presenting to an emergency department. Cardiovascular symptoms were more likely to occur in older patients and patients 50 or 65 years or older were less likely to be dismissed home directly from the emergency department. Moreover, older patients were less likely to be prescribed self-injectable adrenaline. Among possible triggers, food was the less common supposed cause of anaphylaxis in older patients.

A recent epidemiological study investigated trends in anaphylaxis admissions and fatalities by age, sex, and cause in England and Wales over a 20-year period [190]. In patients 60 years of age and older fatalities and hospital admissions were most common for iatrogenic cause and insect sting induced anaphylaxis, while fatal outcomes from food-induced anaphylaxis were most common in the second decade of life and very rare in adults older than 60 years.

In a population-based epidemiologic study conducted in USA using national databases, among various demographic factors, age was most significantly associated with anaphylaxis deaths, with a mortality rate that was highest in people aged between 75 and 84 years (2.04 per million population) and that was lowest in children 17 years or younger, and increased with advancing age [191].

The increased mortality and occurrence of cardiovascular involvement in older patients can be due to a bigger age-related susceptibility to mast cell-derived mediators on cardiovascular system [192], and to the underlying comorbidities, such as coronary diseases. Mast cell-released mediators are in fact able to induce vasospasm of large coronary arteries, a global reduction of myocardial blood flow by influencing the vasomotor tone of small intramural coronary arteries, and may exert direct dysrhythmogenic effects [193]. Data from two thousand allergists treating patients with anaphylaxis in three European countries showed that age was an important risk factor for the onset of circulatory symptoms with a high odds ratio in older patients [194].

Moreover concomitant cardiovascular medications, more often prescribed to patients with advanced age, could negatively influence the outcome. Antihypertensive medication use is associated with increased organ system involvement during anaphylaxis and increased odds of hospital admission, independent of age [195].

It must be also considered that elderly patients are more likely to receive many different medications (such as antibiotics, NSAIDs), particularly parentally administered medications that cause a consequent more rapid exposure of allergen. Brown and collaborators found that, in a large cohort of people admitted to Emergency Departments, older age and drug (both oral and injected) were significantly correlated with hypotensive reactions [196].

There are no absolute contraindications to the prescription of self-injectable adrenaline in older patients at risk of anaphylaxis, however some issues should be considered. The limited mobility or the presence of joint diseases, such as osteoarthritis of the hand, could reduce the ability to use the auto-injector. Also, a negative effect on therapeutically administered adrenaline is exerted by co-administration of cardiovascular medications such as β-blockers, however in patients suffering for heart diseases such as congestive heart failure, the use of β-blockers could improve the survival and the therapeutic benefit can be greater than the risk of aggravating anaphylaxis in some patients [197]. An evaluation of patient on case-by-case basis together with the cardiologist is therefore useful.

### Benefits of physical activity in the elderly with respiratory diseases

In geriatric populations, physical activity represents part of a healthy lifestyle. It favours independence and increases quality of life, improving aerobic capacity, muscle strength, breathing pattern and cardiovascular function, with positive effects on cognitive and psychosocial aspects of daily living in the elderly. Furthermore, the findings from some studies (but not all), support the possibility that exercise may counteract immunosenescence, restoring immune function in older populations and reducing the increased incidence and severity of infectious diseases in senior subjects. It is well known, in fact, that exercise may influence several aspects of immune response, including T-cell phenotype and proliferation, response to vaccination, and cytokine production. However, the underlying mechanisms by which exercise can influence a number of cell types and immune responses still remains to be identified [198, 199].

Physical activity, therefore, seems to play a key role in avoiding the worsening of disabilities, including asthma and other respiratory diseases and may also represent an alternative therapy in patients for whom pharmacological treatment is unavailable, ineffective, or inappropriate. However, even if current data suggests that physical activity may be an effective and logistically easy and clever strategy for counteracting immunosenescence, it is currently underutilized in real clinical settings.

#### Exercise training and effects on immune and respiratory systems

Response to vaccines and novel antigens has been used as a model that may have clinical relevance to understand the role of exercise in modulating immune response. In a study by Smith et al. [200] comparing sedentary and physically active elderly, the physically active group had significantly higher anti-KLH (keyhole-limpet hemocyanin, a protein antigen) IgM, IgG, IgG1, and delayed-type hypersensitivity responses compared with the sedentary older group, showing that regular physical activity in a senior population is associated with a more efficient and robust immune response to novel antigenic challenge [201]. In general, long term exercise interventions seem to show the most promising results, in particular as regards Exercise-related improvements have been reported with respect to antibody titre, T cell function, macrophage response, alterations of the Th1/Th2 cytokine balance, the level of pro-inflammatory cytokines, and changes in naïve/memory cell ratio [200].

Pathophysiologically, in older patients with chronic airflow limitation as a result of asthma, COPD alone or in combination (ACOS), the normal age-related decline in lung function is amplified. Thus, mechanical ventilatory constraints and the perceived respiratory discomfort become the main factors limiting exercise [202].

Therefore, in clinical practice, middle-aged and older adults with moderate/severe persistent asthma are sometimes referred to pulmonary rehabilitation programs (PRP) that often include both breathing exercises and exercise training. Breathing exercises programs may increase muscle strength and are associated with a positive effect on patient health and quality of life [203]. The rationale underlying their participation in these programs is that the training protocol utilized and benefits achieved are similar to those observed in patients with COPD, who represent the majority of individuals referred to PRP [204].

To date, the majority of studies evaluating exercise training in asthma have been performed in children or young adults with mild-to-moderate persistent disease. A Cochrane meta-analysis of 13 randomized controlled trials of exercise training for asthma concluded that training improves cardiopulmonary fitness in the absence of any changes in lung function [205] while a more recent systematic review and meta-analysis of 17 studies and 599 patients [206] found that exercise training was shown to improve asthma symptoms, QoL, exercise capacity, BHR, EIB, and FEV_1_ in asthmatics. In this study, improvements in BHR explained part of the improvement in QoL and exercise capacity.

Another review showed that PRP involving exercise training was effective in improving exercise capacity, muscle force, quality of life and reducing symptoms in patients with COPD and asthma [207].

In patients with COPD and Asthma endurance training is recommended, in the form of continuous and interval training, having similar effects on endurance capacity, executed on either a bike or as walking [208]. However, this recommendation must take into consideration that endurance training may elicit an exercise-induced asthma attack and therefore patients must take the preventive treatments with Beta-2 agonists and a perform warm-up phase of at least 15 min prior to exercise. Strength training is important because of the frequently atrophied skeletal musculature, which triggers the increase of the exercise-induced ventilation by early lactate acidosis and consequently aggravates dyspnoea during exercise. Patients should continue the individualized training after discharge from hospital in the domestic environment, benefitting also of training facilities, patients’ groups and social circumstances. Because of the frequently present cardio-metabolic comorbidities the assessment of the exercise capacity as well as an evaluation of nutrition should be included into a holistic therapeutical approach. In patients with uncontrollable fear of exercise-induced attacks of asthma, additional psychological support should be given [208].

Functional exercise capacity is significantly improved after exercise training, as well as asthma symptoms and quality of life [203]. The efficacy of exercise training programs is demonstrated by a study in which, after a 3-year follow-up of asthmatic patients, that undergone a supervised 10-week exercise training program, a significant decrease in number of emergency room visits was observed. The high compliance rate after the 3 years (68%) showed that, when exercise programs are supervised by health professionals, patients choose to continue to be physically active [209].

To evaluate the outcomes of a PRP, the well-known six-minute walk test is often used as a measure.

As conclusion, a case report interestingly suggests that lifelong sports practice can be a great advantage for the asthmatic elderly [201]. A 86-year old athlete with a lifelong atopic asthma and COPD that competes in swimming and triathlon at an international level, with an advanced obstructive lung disease (FEV1/FVC = 34%), experienced only little perceived respiratory discomfort during exercise and had a peak aerobic capacity (VO2 peak) and cycle work rate in excess of 170% predicted. Therefore, while this case report does not diminish the relative importance of ventilator constraints in dyspnoea or exercise intolerance obstructive lung disease, it shows that the negative consequences of these ventilatory impairments can be attenuated by a constant, lifelong participation in high intensity exercise training with the impressive physiological and psychological adaptations associated.

Physical activity should be recommended as a supplementary therapy to medication [210]. Strategies to increase physical and sports activity participation among older people should include (i) raising awareness of the benefits and minimize the perceived risks of physical activity and (ii) improving the environmental and financial access to community-based exercise programs such as may exist for middle-aged and older individuals with other chronic condition [211]. Given this evidence, an increase in the use of physical activity programs by the healthcare community may result in improved health of geriatric populations [198]. If positive effects will also be confirmed in terms of counteraction to immunosenescence, exercise could be a highly cost-effective measure to improve human quality of life compared with other strategies currently being pursued.

## Conclusions

The mechanisms that allow “successful” aging are related both to psychophysical conditions and to the immune system behavior. In this regard, in addition to the precise knowledge of the immunological mechanisms in geriatric age, of the lymphoproliferative responses and of the ROS production, the lifestyles, the diet and the physical activity should also be analysed.

Allergic diseases are often thought to be specific of childhood and youth, however, these conditions often persist into older age and can occasionally appear in the elderly. Several are the factors that make more complicated the diagnosis and the management of allergic diseases in aged people, such as frailty, comorbidities and multiple concomitant medications. At the same time, nutritional deficiencies such as vitamin D deficiency, genetic factors, hormonal imbalances, or inflammaging may affect the immune system, causing alterations of the responses of immunocompetent cells. All parameters along with the management approach proposed for diseases can act as support to improve the psychophysical conditions of people over 65 and ensure an active and healthy aging.

## Abbreviations

NK: natural killer
CD: cluster of differentiation
TREC: T cell receptor rearrangement excision circles
IL: interleukin
IL-7R: interleukin 7 receptor
CMV: cytomegalovirus
IRP: immune risk profile
TCC: T cell clones
Ig: immunoglobulin
TNF: tumor necrosis factor
IFN: interferon
IL-18BP: IL-18 binding protein
TLR: Toll-like receptor
NYHA: New York Heart Association
PBMC: peripheral blood mononuclear cell
IL-1Ra: IL-1 receptor antagonist
TGF: transforming growth factor
WHO: World Health Organization
PM: particulate matter
QoL: quality of life
COPD: chronic obstructive pulmonary disease
SAC: seasonal allergic conjunctivitis
PAC: perennial allergic conjunctivitis
VKC: vernal keratoconjunctivitis
AKC: atopic keratoconjunctivitis
ABC: atopic blepharoconjunctivitis
GPC: giant papillary conjunctivitis
CAC: chronic allergic conjunctivitis
Th: T helper
TNF: tumor necrosis factor
AIT: allergen (specific) immunotherapy
SCIT: subcutaneous immunotherapy
SLIT: sublingual immunotherapy
EMA: European Medicines Agency
FDA: Food and Drug Administration
Treg: T regulatory
ACE: angiotensin converting enzyme
ACD: allergic contact dermatitis
AD: atopic dermatitis
CU: chronic urticaria
EAACI: European Academy of Allergy and Clinical Immunology
GA2LEN: Global Allergy and Asthma European Network
EDF: European Dermatology Forum
WAO: World Allergy Organization
CSU: chronic spontaneous urticaria
ASST: autologous serum skin test
MGUS: monoclonal gammopathy of undetermined significance
HAE-C1-INH: hereditary angioedemaC1 inhibitor
AAE-C1-INH: acquired angioedemaC1 inhibitor
AE-ACEi: angioedema–angiotensin converting enzyme inhibitor
CNS: central nervous system
CYP: cytochromes P
SPT: skin prick tests
FA: food allergy
S-IgA: secretory antigen-specific IgA
TJ: tight junction
ADRs: adverse drug reactions
DA: drug allergy
HLA: human leucocyte antigen
DiHS: drug-induced hypersensitivity syndrome
DRESS: drug rash with eosinophilia and systemic symptoms
CI: confidence interval
NSAIDs: non-steroidal anti-inflammatory drugs
GIFA: Gruppo Italiano di Farmacoepidemiologia nell’anziano
EDs: Emergency Departments
KLH: keyhole-limpet hemocyanin
ACOS: asthma-COPD overlap syndrome
PRP: pulmonary rehabilitation programs
BHR: bronchial hyperresponsiveness
EIB: exercise-induced bronchoconstriction
FEV: forced expiratory volume
FVC: forced vital capacity
ROS: reactive oxygen species
